# *Lacticaseibacillus casei* Zhang modulates iron homeostasis in iron-overloaded mice via an IL-22-dependent pathways

**DOI:** 10.1038/s41538-026-00899-0

**Published:** 2026-05-26

**Authors:** Feiyan Zhao, Da Ma, Yinyin Gao, Luping Yang, Lu Gao, Zhihong Sun

**Affiliations:** 1 Key Laboratory of Dairy Products Processing, Ministry of Agriculture, Inner Mongolia Agricultural University, Hohhot, PR China; 2 Key Laboratory of Dairy Biotechnology and Engineering, Ministry of Education, Inner Mongolia Agricultural University, Hohhot, PR China; 3 School of Food & Pharmaceutical Science and Technology, Guangzhou College of Technology and Business, Foshan, PR China

**Keywords:** Diseases, Immunology, Microbiology

## Abstract

This study elucidated the role of IL-22 in the disruption of iron homeostasis and gut microbiota dysbiosis induced by a high-iron diet (HI) and evaluated the therapeutic potential of the probiotic *Lacticaseibacillus casei Zhang* (LCZ). In mice fed an HI diet, HI increased serum iron (SI) and IL-22 levels and reduced total iron-binding capacity (TIBC), accompanied by the enrichment of opportunistic taxa, including *Parabacteroides goldsteinii*, and functional metabolic disturbances, including increased lithocholic acid levels and suppression of lactose degradation pathways. LCZ intervention attenuated these alterations by partially restoring TIBC, reducing IL-22 levels, and increasing the relative abundance of taxa commonly linked to gut health, including *Bifidobacterium animalis*. In contrast, IL-22 knockout (KO) mice did not exhibit LCZ-associated improvement in iron indices, supporting the requirement for IL-22 to mediate probiotic responsiveness in iron overload. Multi-omics analyses further indicated that LCZ mitigated HI-associated bile acid dysregulation and impaired glucose metabolism, whereas KO mice exhibited more pronounced metabolic disruption. Collectively, these findings identified IL-22 as a key immune node associated with the dietary iron-microbiota-host axis and supported targeting the IL-22-linked pathway as a potential strategy to reduce iron overload-associated risks.

**Figure:**
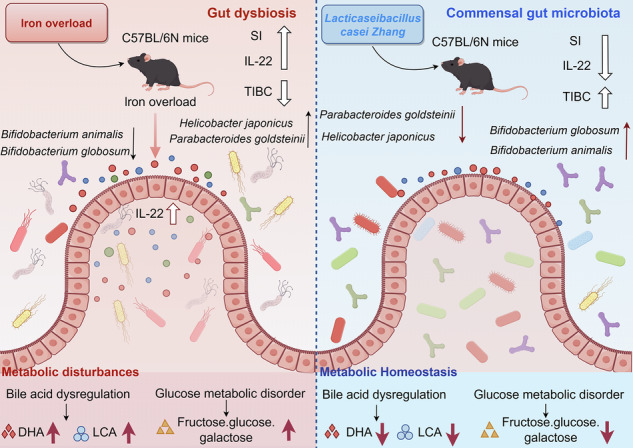

## Introduction

Iron is an essential trace element involved in oxygen transport, electron transfer, and DNA synthesis^1^. However, as a transition metal, iron can also catalyze the Fenton and Haber-Weiss reactions, generating reactive oxygen species (ROS) that cause oxidative damage to cellular components^2^. With the rapid expansion of the modern food industry and increased public awareness of nutrition, iron supplements, iron-fortified foods, and iron-enriched health products have become increasingly available and diversified in consumer markets worldwide. According to the Global Burden of Disease Study, between 1990 and 2019, the average daily dietary iron intake among Chinese adults increased from ~26 to 30 mg in men and from ~23 to 27 mg in women^3^. In the United States, ~14–18% of the population consumes iron supplements, with the highest prevalence observed among pregnant (72%) and lactating (60%) women. However, excessive iron intake has been shown to confer substantial health risks, including hyperferritinemia^4^ and hepatic injury^5^, and has been implicated in the development of type 2 diabetes^6^. In contrast, insufficient iron intake results in adverse clinical outcomes, most notably iron deficiency anemia^7^. Iron is a critical determinant of bacterial proliferation^8^. Consequently, the maintenance of iron homeostasis is of central importance to host health.

The gut microbiota, often referred to as the host’s “second genome,” exerts a profound influence on overall health by maintaining the microbial community structure and functional stability^9^. Accumulating evidence indicates that high-iron conditions markedly disrupt intestinal microbial homeostasis, resulting in dysbiosis^10,11^. For example, Derman et al. demonstrated in murine models that iron supplementation during post-antibiotic recovery promoted a shift toward a microbiota with pro-carcinogenic features^12^. Clinical investigations have further revealed a dose-dependent relationship between elevated iron intake and alterations in gut microbial composition, characterized by an increased abundance of Proteobacteria and a simultaneous depletion of beneficial taxa^13^. Such dysbiosis not only exacerbates intestinal inflammation but may also potentiate systemic disease processes associated with iron overload through complex microbiota-host interactions. Probiotics modulate the gut microbiota, enhance intestinal barrier function, and regulate immune responses^14,15^. While previous studies have demonstrated the efficacy of specific probiotic strains in alleviating iron deficiency anemia, their role in the context of iron overload remains largely unexplored^16,17^. Nevertheless, current research has largely focused on the benefits of probiotics under iron-deficient conditions, leaving substantial gaps in our understanding of how probiotics modulate gut microbial structure and function to counteract the adverse effects of excessive iron intake in high-iron dietary conditions. In particular, the extent to which these effects are mediated by or dependent on host immune responses, including key cytokines, remains unclear. Therefore, clarifying the dynamic interactions among high-iron diets, host immunity, and gut microbiota is essential for elucidating the mechanisms underlying iron overload-associated pathologies and developing effective intervention strategies.

To address these unresolved questions, this study selected *Lacticaseibacillus casei Zhang* (LCZ), a probiotic strain isolated from traditional fermented mare’s milk in Inner Mongolia that exhibits robust gastrointestinal tolerance and colonization capacity^18,19^, as a candidate modulator of iron metabolism. The investigation focused on three central objectives: first, to define the effects of a high-iron diet (HID) on systemic iron homeostasis, gut microbiota composition and function, expression of key immune factors such as IL-22, and inflammatory status; second, to elucidate the regulatory role of the host immune factor IL-22 in mediating HID-associated microbiota-host crosstalk and its impact on microbial community structure and function; and third, to determine whether LCZ intervention could ameliorate HID-induced dysbiosis, immune dysregulation, and associated health impairments, as well as clarify the underlying mechanisms and conditions required for efficacy.

In this study, an integrated experimental strategy combining metagenomic deep sequencing, immune factor screening arrays, and CRISPR/Cas9-generated IL-22 knockout (KO) mice was employed to systematically delineate the tripartite interaction network among HID, host immunity with a focus on IL-22, and the gut microbiota. These findings not only identified potential microbial biomarkers but also highlighted the therapeutic potential of LCZ in iron metabolism disorders. Collectively, these results provide novel insights into immune factor-mediated microbiota-host interactions under iron overload conditions and establish a scientific basis for developing probiotic-based precision nutrition strategies aimed at mitigating iron overload-associated health risks.

## Results

### Effects of LCZ intervention on serum iron-related biochemical indices and organ coefficients

**Fig. 1:**
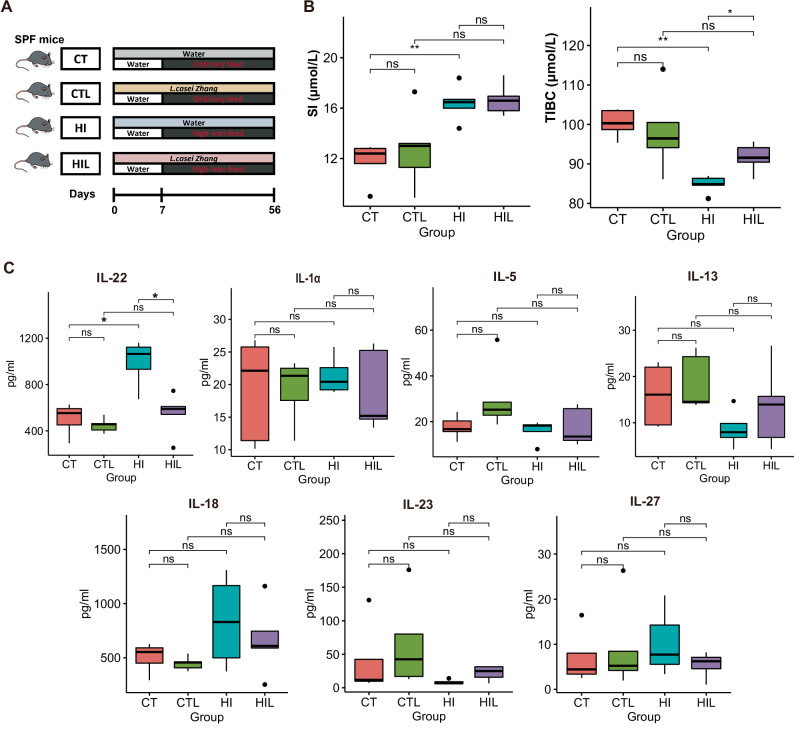
Effect of LCZ intervention on serum iron and immune factors in a high-iron diet. **A** Experimental design of the study. **B** Serum iron (SI) and total iron-binding capacity (TIBC). **C** Immune factors: IL-22, IL-1α, IL-5, IL-13, IL-18, 1L-23, and IL-27. *P < 0.05, **P < 0.01, *** *P* < 0.001, ****P < 0.0001. ns non-significant.

To evaluate the impact of dietary iron loading on systemic iron homeostasis, SI and TIBC levels were measured. As shown in Fig. 1B, the high-iron diet significantly increased SI (CT vs HI: *P* < 0.01; CT: 11.74 ± 1.61 μmol/L vs HI: 17.00 ± 2.62 μmol/L) and markedly decreased TIBC (CT vs HI: *P* < 0.01; CT: 100.34 ± 3.52 μmol/L vs HI: 84.39 ± 1.50 μmol/L). Under high iron conditions, LCZ treatment significantly increased TIBC relative to the HI group (HI vs. HIL: *P* < 0.05; HI: 84.39 ± 1.50 μmol/L vs. HIL: 94.77 ± 2.00 μmol/L), but did not significantly alter SI (*P* > 0.05). LCZ supplementation in the control diet did not significantly affect SI or TIBC compared to the CT group (*P* > 0.05). Liquid-phase suspension array analysis of serum immune factors identified IL-22 as the only cytokine that differed significantly among the groups (*P* < 0.05) (Fig. 1C). Mice fed the high-iron diet exhibited higher serum IL-22 concentrations than controls (CT vs HI: *P* < 0.05; CT: 503.37 ± 134.65 pg/mL vs HI: 990.76 ± 219.46 pg/mL), whereas LCZ treatment significantly attenuated this increase (HI vs HIL: *P* < 0.05; HI: 990.76 ± 219.46 pg/mL vs HIL: 548.42 ± 181.21 pg/mL). No significant differences were observed in the liver or spleen organ coefficients between the groups (Supplementary Fig. S1).

### Effect of LCZ intervention on intestinal microbial structure in mice fed a high-iron diet

**Fig. 2:**
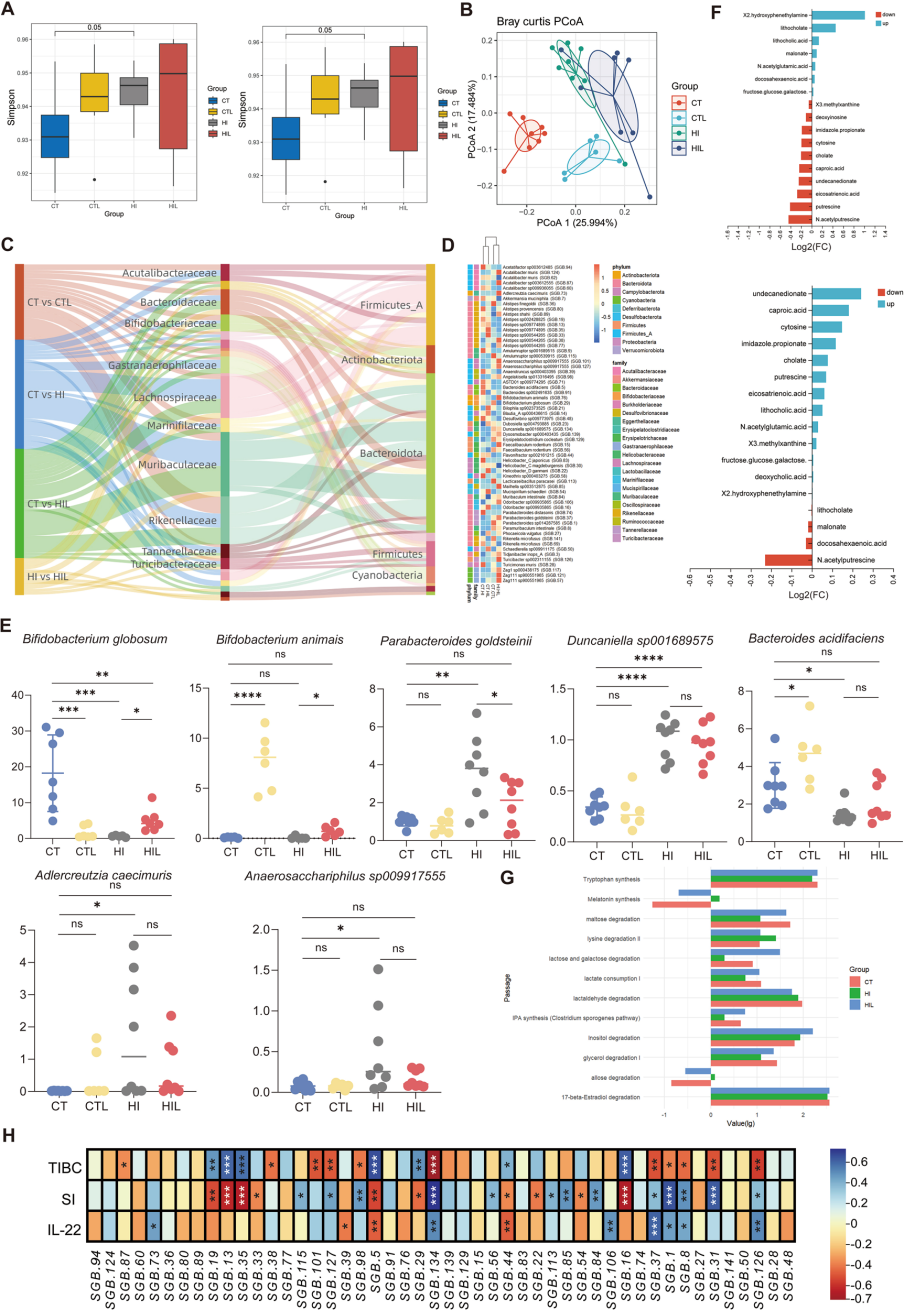
Impact of LCZ intervention on gut microbial composition and metabolic profiles in mice fed a high-iron diet. **A** Simpson and Shannon indices of each group. **B** Principal coordinate analysis (PCoA, Bray-Curtis distance) revealed differences in the gut microbial metagenomes between groups. **C** Sankey diagram illustrating taxonomic composition from phylum to family level. Taxonomic ranks are indicated by prefixes: p_ (phylum) and f_ (family). **D** Family level bacterial taxonomic profile displayed using heatmap analysis. **E** Species-resolved genome bins (SGBs) showing differential abundance across groups pre- and post-intervention (Wilcoxon test; error bars: SD). **F** Predicted metabolite profiles exhibit significant upregulation/downregulation across experimental groups: upper panel shows CT vs HI comparison, lower panel displays HI vs CTL analysis. **G** Predicted metabolic pathway analysis revealed key degradation/biosynthetic pathways and their associated regulatory networks. **H** Correlations of Gut Microbial Species-Level Genomes with Iron Metabolism Parameters (TIBC, SI) and Inflammatory Cytokine IL-22. *P < 0.05, **P < 0.01, *** *P* < 0.001, ****P < 0.0001. ns non-significant.

To characterize the regulatory effects of LCZ on the gut microbiota under high-iron conditions, the microbial community structures were compared across groups. LCZ supplementation alone did not significantly affect alpha diversity relative to the CT group, as indicated by the Shannon and Simpson indices (*P* > 0.05; Fig. 2A). In contrast, HI feeding significantly increased α-diversity compared to the CT control (*P* < 0.05; Fig. 2A). PCoA demonstrated a clear separation between the HI and CT groups, indicating a substantial shift in the community structure. LCZ intervention under high-iron conditions further reshaped the altered microbiota, with HIL samples distinctly separated from the HI group (Fig. 2B), consistent with a remodeled community organization under iron overloading.

Sankey diagrams were generated to visualize the taxonomic redistribution between high-iron and control conditions and depict dominant flow patterns from phylum to family levels (Fig. 2C). Compared with the CT controls, the HI diet altered the microbial composition, including changes in Lachnospiraceae (Firmicutes_A), Muribaculaceae (Bacteroidota), and Rikenellaceae (Bacteroidota). At the species level, the HI group exhibited enrichment of taxa including *Helicobacter_C japonicus* (SGB.83), *Desulfovibrio sp009773975* (SGB.48), *Parabacteroides goldsteinii* (SGB.37), *Anaerotruncus sp000403395* (SGB.39), and *Bacteroides sp002491635* (SGB.91). These increases were attenuated by LCZ treatment. In parallel, LCZ supplementation increased the relative abundance of multiple taxa, including *Bifidobacterium globosum* (SGB.29), *Bifidobacterium animalis* (SGB.76), *Acutalibacter muris* (SGB.124), *Acutalibacter sp003612555* (SGB.87), *Adlercreutzia caecimuris* (SGB.73), and *Faecalibaculum rodentium* (SGB.15) (Fig. 2D, E). Collectively, these results indicate that LCZ reshapes gut microbial communities under iron overload.

Pearson’s correlation analysis further supported the association between microbial taxa and immune or iron homeostasis parameters (Fig. 2H). Several taxa, including *Bifidobacterium globosum* (SGB.29), *Bifidobacterium animalis* (SGB.76), *Alistipes sp009774895* (SGB.13), *Alistipes sp002428825* (SGB.19), *Bacteroides acidifaciens* (SGB.5), and *Odoribacter sp009935865* (SGB.16), were positively correlated with TIBC and negatively correlated with IL-22 and SI. In contrast, taxa including *Turicibacter sp002311155* (SGB.126), *Duncaniella sp001689575* (SGB.134), *Adlercreutzia caecimuris* (SGB.73), *Anaerosacchariphilus* sp009917555 (SGB.101), *Parabacteroides goldsteinii* (SGB.37), *Parabacteroides sp014287585* (SGB.1), and *Paramuribaculum intestinale* (SGB.8) showed the opposite pattern, with positive correlations with IL-22 and SI and negative correlations with TIBC. These associations were consistent with the coordinated variations among the iron indices, immune signaling, and microbial composition.

To explore the functional shifts associated with high-iron feeding and LCZ intervention, metabolite profiles were inferred based on functional predictions from the metagenomic data (Fig. 2F). Relative to the CT group, the HI group exhibited higher predicted levels of X2-hydroxyphenethylamine, lithocholate, lithocholic acid, malonate, N-acetylglutamic acid, docosahexaenoic acid, and fructose/glucose/galactose-related metabolites, whereas the predicted levels of caproic acid, undecanedionate, imidazole propionate, and cholate were reduced. Pathway analysis identified three significantly upregulated and six downregulated pathways in the HI group compared to the CT group (Fig. 2G). The downregulated pathways included tryptophan biosynthesis (amino acid metabolism), maltose degradation II (glycan metabolism), lactose/galactose degradation (carbohydrate metabolism), lactate consumption I (glycolysis/gluconeogenesis), IPA synthesis (Clostridium sporulation pathway), and glycerol degradation I (lipid metabolism). In contrast, maltose degradation (glycan metabolism), lysine degradation II (amino acid catabolism), and cellulose degradation (rare sugar metabolism) were all upregulated. LCZ intervention largely restored these pathway alterations to levels observed in the CT group, indicating the modulation of the microbial functional potential under high-iron conditions.

### Effects of IL-22 gene deletion on gut microbial diversity and composition in mice

**Fig. 3:**
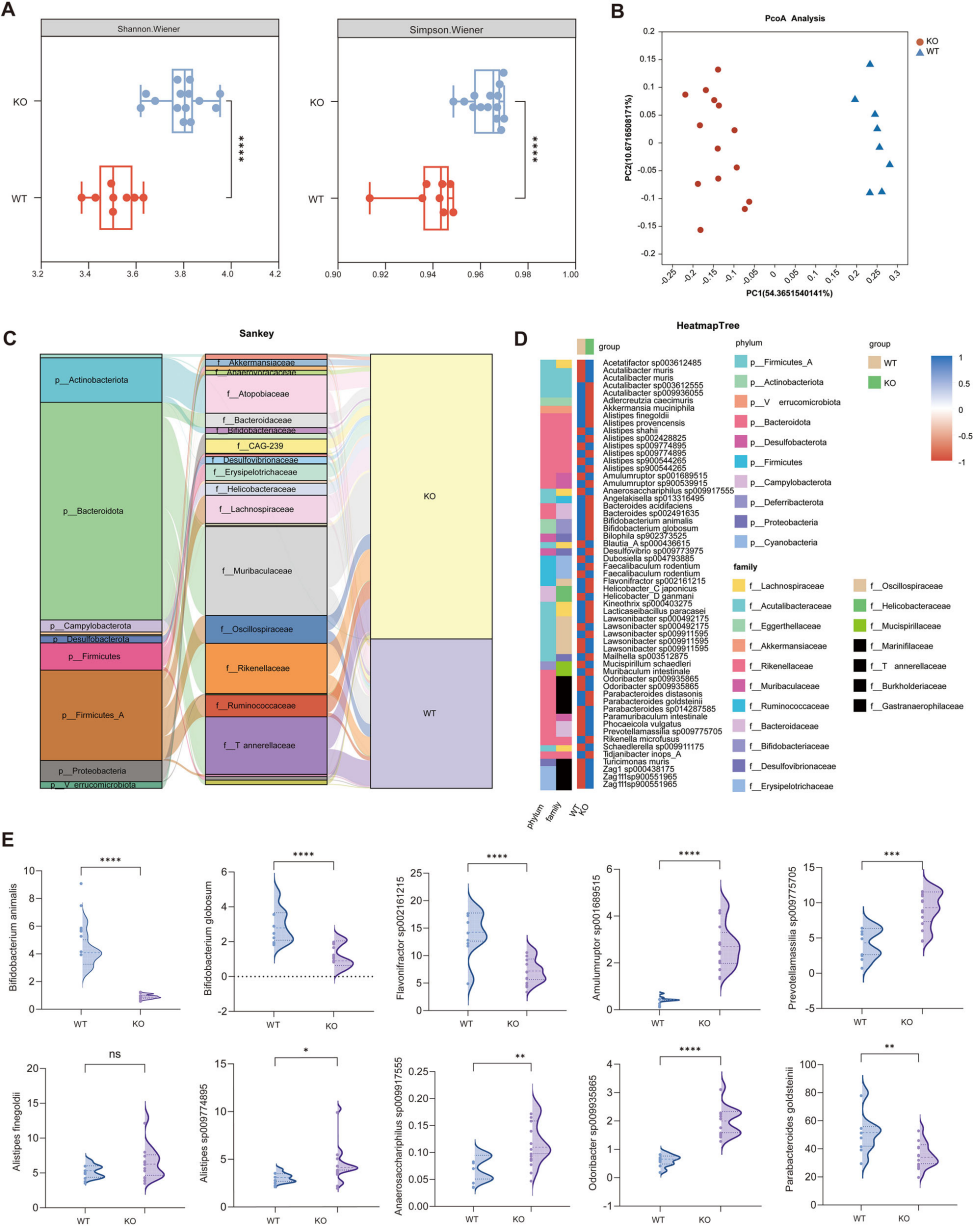
Baseline gut microbiota profiling reveals distinct diversity and community structure in IL-22 Deficient Versus Wild-Type Mice. **A** Simpson index and Shannon index of each group. **B** PCoA reveals structural divergence of gut microbiota in IL-22-deficient versus wild-type mice. **C** Sankey diagram illustrating taxonomic flow and associations between bacterial phyla and families. taxonomic ranks are indicated by prefixes: p_ (phylum) and f_ (family). **D** Species-level bacterial community structure visualized using heatmap clustering. **E** Differentially abundant bacterial strains (species-level genome bins) in IL-22 deficient versus wild-type mice under homeostatic conditions. *P < 0.05, **P < 0.01, *** *P* < 0.001, ****P < 0.0001. ns non-significant.

To delineate the intrinsic effects of IL-22 deficiency on gut microbiota composition, baseline metagenomic sequencing was performed in 8-week-old IL-22 knockout (KO) and corresponding wild-type (WT) mice maintained on a standard chow diet without additional experimental interventions. The α-diversity differed significantly among genotypes (*P* < 0.05) (Fig. 3A). PCoA based on Bray-Curtis distances further demonstrated clear separation of microbial community structure between WT and KO mice (PC1:54.37% variance; PC2:10.67% variance) (Fig. 3B), indicating that IL‑22 deletion alone was sufficient to induce substantial remodeling of fecal microbiota architecture under identical dietary conditions. Consistent with this pattern, Sankey flow diagrams showed a pronounced compositional divergence between the WT and KO groups (Fig. 3C), and heatmap profiling further delineated the genotype-dependent global community structure (Fig. 3D). At the species level, several genotype-discriminatory signatures were identified. KO mice exhibited significant depletion of *Bifidobacterium globosum* (SGB.29) and *Bifidobacterium animalis* (SGB.76) (*P* < 0.05), whereas *Alistipes provencensis* (SGB.80), *Prevotellamassilia sp009775705* (SGB.31), and *Amulumruptor sp001689515* (SGB.9) were significantly enriched (*P* < 0.05) (Fig. 3E). Notably, Bacteroidetes was the dominant phylum in both genotypes. Collectively, these findings indicated that IL‑22 deletion selectively reshapes the gut microbial ecosystem.

### Effects of LCZ on iron metabolism and microbial ecology in IL-22-deficient mice

**Fig. 4:**
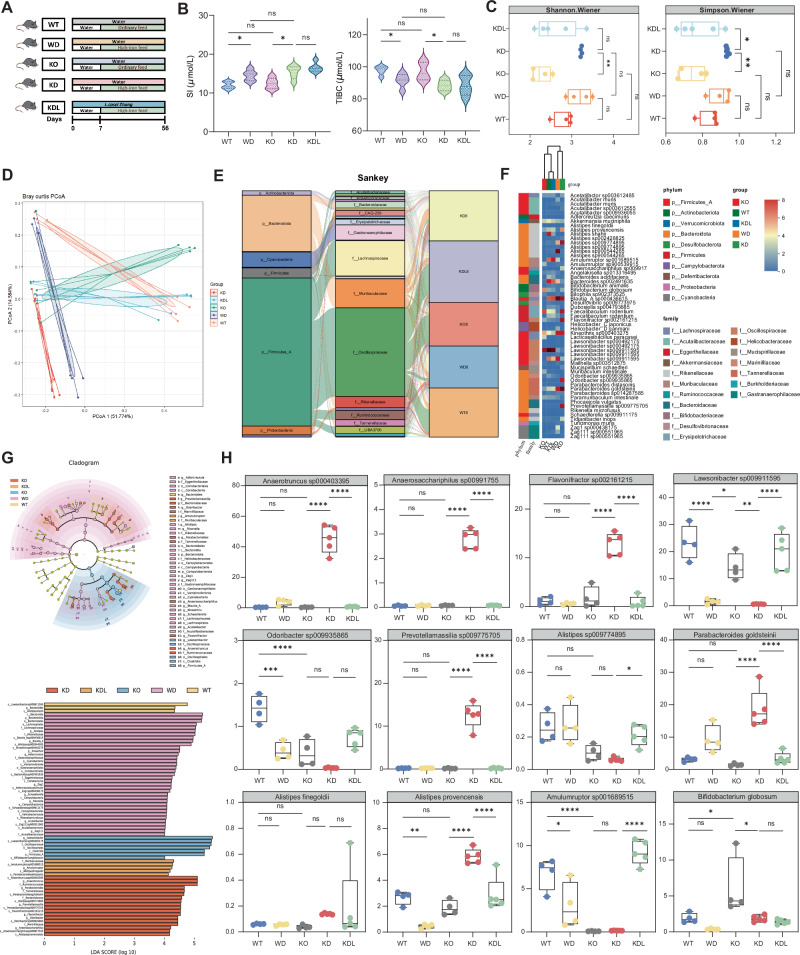
Microbiome-targeted iron homeostasis restoration by *L. casei Zhang* in IL-22 knockout mice. **A** Experimental design. **B** Serum iron (SI) and total iron binding capacity (TIBC). **C** Simpson and Shannon indices of each group. **D** Principal coordinate analysis (PCoA, Bray-Curtis distance) revealed differences in the gut microbial metagenome between the groups. **E** Sankey diagram illustrating taxonomic flow and associations between bacterial phyla and families. taxonomic ranks are indicated by prefixes: p_ (phylum) and f_ (family). **F** Species-level bacterial community structure visualized using heatmap clustering. **G** Phylogenetic cladogram generated by LEfSe analysis illustrating differentially abundant microbial taxa among experimental groups (LDA score > 4.0, *P* < 0.05). Taxonomic ranks are indicated by prefixes: p_ (phylum), c_ (class), o_ (order), f_ (family), g_ (genus), and s_ (species). Each node represents a distinct microbial taxon, with node size proportional to its relative abundance. Colors denote enrichment across groups: red, enriched in the KD group; orange, enriched in the KDL group; blue, enriched in the KO group; pink, enriched in the WD group; and yellow, enriched in the WT group. **H** Differential abundance of bacterial strains (species-level genome bins) between IL-22-deficient and wild-type mice after an 8-week intervention. *P < 0.05, **P < 0.01, *** *P* < 0.001, ****P < 0.0001. ns non-significant.

To determine whether IL-22 functions as a central mediator linking systemic iron homeostasis to gut microbial dysbiosis under high-iron dietary conditions, wild-type and IL-22 knockout mice were examined (Fig. 4A). Consistent with the findings in C57BL/6 N mice, WT animals fed the high iron diet exhibited increased SI (WD vs. WT: 14.52 ± 1.40 vs. 11.98 ± 0.93 μmol/L; *P* < 0.05) and reduced TIBC (91.49 ± 4.32 vs. 102.34 ± 3.85 μmol/L; *P* < 0.05) levels. KO mice showed a similar response, with the KD group showing a higher SI (15.34 ± 1.69 vs. 12.44 ± 1.42 μmol/L) and lower TIBC (88.34 ± 3.44 vs. 96.98 ± 5.67 μmol/L) than the KO control group (*P* < 0.05; Fig. 4B). Notably, LCZ intervention in KO mice (KDL group) did not significantly alter SI or TIBC compared to the KD group (*P* > 0.05), indicating that IL-22 was required for the LCZ-associated effects on iron-related indices.

After 8 weeks of dietary and LCZ interventions, metagenomic profiling of fecal samples revealed distinct community-level patterns (Fig. 4C). In WT mice, α-diversity did not differ significantly between groups, whereas the KD group exhibited significantly increased diversity compared to the KO controls (*P* < 0.05). The PCoA showed a clear separation of the HID-fed WD and KD clusters from the WT, KO, and KDL groups (Fig. 4D). Sankey analysis indicated that Firmicutes, particularly Oscillospiraceae and Lachnospiraceae, together with Bacteroidota dominated by Muribaculaceae, were the major lineages associated with WT/KO/KDL cohorts (Fig. 4E). Baseline profiling indicated that both genotypes clustered primarily within the Bacteroidota lineages, including the Muribaculaceae, Rikenellaceae, and Tannerellaceae. Following intervention, Firmicutes were selectively enriched in WT/KO/KDL groups, whereas Bacteroidota predominated in HID-fed WD and KD mice.

LEfSe (LDA effect size) analysis was then used to identify taxa that differed significantly among groups, with phylogenetic cladograms summarizing the hierarchy of discriminant features (LDA score >4). WT mice were characterized by the enrichment of *Lawsonibacter sp009911595* (SGB.64) and *Alistipes shahii* (SGB.89). In contrast, the WD group showed increased abundance of *Blautia_A sp000436615* (SGB.14), *Alistipes sp900544265* (SGB.33), *Kineothrix sp000403275* (SGB.58), *Bacteroides sp002491635* (SGB.91), *Adlercreutzia caecimuris* (SGB.73), *Schaedlerella sp009911175* (SGB.50), *Rikenella microfusus* (SGB.69), and *Acutalibacter muris* (SGB.124). KO mice were distinguished by *Lawsonibacter sp000492175* (SGB.64) and *Bifidobacterium globosum* (SGB.29), whereas the KD group showed selective enrichment of *Anaerosacchariphilus sp009917555* (SGB.101), *Odoribacter sp009935865* (SGB.16), *Alistipes sp009774895* (SGB.13), *Parabacteroides goldsteinii* (SGB.37), *Flavonifractor sp002161215* (SGB.44), and *Alistipes provencensis* (SGB.80) (Fig. 4F–H). Collectively, these findings indicate that dietary iron overload was associated with pronounced shifts in gut microbial composition and that LCZ intervention restored the abundance of multiple taxa in WT mice but not in IL-22-deficient mice.

### Effects of LCZ intervention on high-iron diet-induced metabolic dysregulation in IL-22 knockout mice

**Fig. 5:**
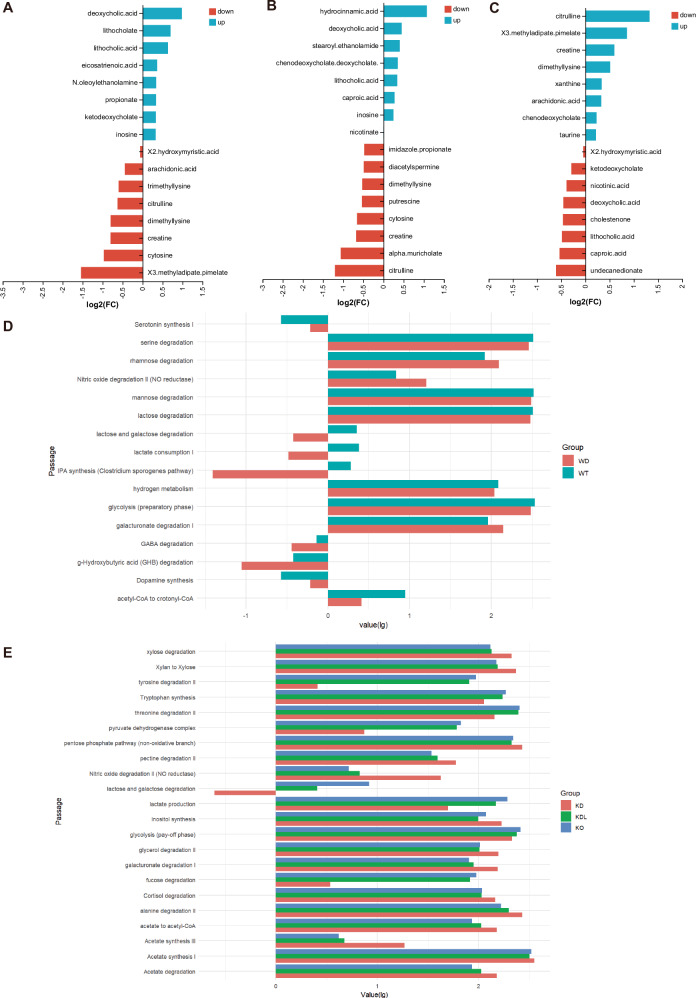
Modulatory effects of LCZ intervention on high iron diet-induced metabolic and pathway perturbations in IL-22 knockout mice. **A** Compared to the WT controls, the WD group exhibited significantly upregulated and downregulated metabolites. **B** Compared to the KO controls, the KD group exhibited significantly upregulated and downregulated metabolites. **C** Compared to the KD controls, the KDL group exhibited significantly upregulated and downregulated metabolites. **D** Metabolic pathway analysis identified critical degradation/biosynthesis pathways that were differentially regulated between the WT and WD groups. **E** Metabolic pathway analysis identified critical degradation/biosynthesis pathways that were differentially regulated between the KO/KD and KDL groups.

Based on metagenomic functional predictions, diet- and genotype-dependent differences in metabolic pathways were quantified using log2 fold-change (log2(FC)) analyses. Compared to the WT controls, the WD group exhibited significant activation of bile acid metabolism (*P* < 0.05), accompanied by higher predicted levels of secondary bile acids, including deoxycholic and lithocholic acids. In parallel, the WD group showed increased predicted levels of nucleoside metabolites (inosine) and fatty acid derivatives (N-oleoylethanolamine), along with marked reductions in predicted intermediates linked to energy metabolism, including TCA cycle-associated X3-methyladipate/pimelate and creatine (Fig. 5A).

In IL-22 knockout mice, the KD group displayed elevated predicted levels of aromatic fatty acids (hydrocinnamic acid), short-chain fatty acids (caproic acid), and nicotinate (*P* < 0.05), whereas the predicted levels of key intermediates related to the urea cycle (citrulline) and microbiota-derived metabolism (imidazole propionate) were reduced (Fig. 5B). In contrast, the probiotic-supplemented KDL group exhibited a metabolic profile that differed from the KD group, with an increase in lithocholic acid, as reflected by lower predicted levels of cholestenone and ketodeoxycholate (Fig. 5C). These patterns indicate that IL-22 deficiency is associated with altered host-microbiota metabolic crosstalk, particularly involving bile acid and amino acid pathways, and modifies the metabolic response to dietary iron.

Pathway-level analyses were consistent with these results, showing significant differences between the WD and WT groups, particularly in lactose/galactose degradation, lactate consumption I, and IPA synthesis (Clostridium sporogenes pathway) (Fig. 5D). In IL-22 knockout mice, broad pathway dysregulation was observed, with the KD group showing downregulation of lactose/galactose degradation, tyrosine degradation II, tryptophan synthesis, and fucose degradation, and upregulation of xylose degradation, xylan-to-xylose conversion, nitric oxide degradation II (NO reductase), and acetate synthesis III relative to KO controls (Fig. 5E). LCZ intervention in KO mice (KDL group) largely restored these pathway alterations to the KO baseline levels.

## Discussion

Iron is an essential mineral that participates in various biological processes^20^. However, excessive iron intake poses clinically relevant risks by promoting systemic iron overload and inducing adverse effects in the intestinal tract and liver, while also perturbing the gut microbiota^21^. Oral iron supplementation has been reported to precipitate gut microbial dysbiosis and increase disease susceptibility, complicating the development of population-level recommendations for iron intake^22,23^. Although selected probiotic strains, such as *Lactiplantibacillus plantarum* 299 v, have been shown to enhance iron absorption, the existing literature has predominantly focused on iron-deficient states^24^. In contrast, the interplay between host immunity and gut microorganisms under iron overload and the potential for probiotic intervention in this setting remain insufficiently characterized. Accordingly, the present study evaluated the effects of LCZ in an iron-overload model and clarified the contribution of gut microbiota and host immune signaling to iron-overload-associated disturbances.

In this study, high-iron diet feeding was associated with increased SI, reduced TIBC, and a pronounced elevation of serum IL-22. IL-22, a cytokine produced primarily by type 3 innate lymphoid cells (ILC3s) and Th17 cells, has been implicated in intestinal barrier maintenance and the regulation of antimicrobial peptide expression^25^. However, excessive IL-22 signaling has also been linked to inflammatory disorders^26^. The concurrent elevation of IL-22 with SI accumulation and TIBC reduction in the HI group suggests that heightened IL-22 signaling may contribute to dysregulated intestinal iron handling. Notably, LCZ intervention attenuated the HI-associated increase in IL-22 and partially restored TIBC, whereas LCZ did not significantly alter IL-22 levels in healthy controls, supporting this context-dependent effect. Importantly, in IL-22 knockout mice, neither high iron feeding nor LCZ intervention significantly modified SI or TIBC, providing genetic evidence consistent with the requirement of IL-22 in LCZ-associated modulation of iron-related indices and mitigation of iron overload-associated dysregulation.

The gut microbiota was evaluated to determine whether LCZ alters microbial ecology under physiological and iron-overload conditions. LCZ intervention did not significantly change the relative abundance of major phyla or genera, nor did it alter β-diversity compared to the CT controls, indicating that LCZ induced minimal perturbation of the gut microbiota in a healthy state. In contrast, the HI group exhibited pronounced community shifts, consistent with the possibility that dietary iron overload alters intestinal niche conditions and promotes an ecological imbalance. Specifically, the HI group showed enrichment of *Helicobacter_C japonicus* (SGB.83), *Desulfovibrio sp009773975* (SGB.48), and opportunistic taxa such as *Parabacteroides goldsteinii* (SGB.37) and *Adlercreutzia caecimuris* (SGB.73), aligning with prior reports of iron-associated *Parabacteroides* expansion^27^. LCZ treatment increased the abundance of taxa, including *Bifidobacterium animalis* (SGB.76) and *Bifidobacterium globosum* (SGB.29). These taxa have been previously reported to be associated with SCFA production and barrier-supportive functions^28–30^, consistent with the potential health benefits observed in the present study. Consistent with iron fortification studies reporting increased Enterobacteriaceae and Bacteroides with concomitant reductions in *Lactobacillus*^23,31^, *Bifidobacterium animali* has been characterized as a barrier-associated species that supports intestinal integrity and limits pathogen colonization^32^. Given iron’s established role in supporting pathogenic bacterial growth and virulence^33^, these findings suggest that dietary iron facilitates the expansion of pathobionts, potentially accompanied by the suppression of *Bifidobacterium* through competitive niche dynamics. Collectively, these data indicate that excess dietary iron disrupts colonic microbial equilibrium, resulting in a relative increase in taxa with higher pathogenic potential and a decrease in barrier-associated strains. Correlation analyses further supported these relationships, with taxa such as *Bifidobacterium* showing positive associations with TIBC but negative associations with SI and IL-22, whereas *Parabacteroides goldsteinii* demonstrated the opposite pattern, showing positive associations with elevated SI and IL-22 and taxa enriched under iron overload conditions. This network was consistent with a high-iron diet, selecting a pro-inflammatory microbiota configuration, whereas LCZ-associated benefits coincided with shifts toward taxa more commonly linked to intestinal health.

To clarify the interplay between gut microbiota and host immunity under iron overload, IL-22 knockout mice were used. Baseline profiling at week 0 demonstrated significant differences between IL-22 knockout and wild-type mice in early adulthood, including lower abundances of *Bifidobacterium globosum* (SGB.29) and *Bifidobacterium animalis* (SGB.76) in KO mice, supporting the evidence that host genetics contribute to the gut microbial composition^34^. Following high iron intake, both WT and KO mice showed significant microbiota alterations compared to their corresponding controls. Notably, HID-fed KO mice exhibited increased α-diversity. A high-iron diet induces gut microbial ecological disarray, characterized by the expansion of opportunistic pathogens (e.g., *Parabacteroides goldsteinii*) and suppression of beneficial taxa. This shift in microbial composition, accompanied by increased α-diversity, reflects a state of niche disruption and pathological diversification, rather than a healthy increase in microbial richness. This pattern was not observed in the WT mice. This genotype-dependent response implicates IL-22 as an important regulator of microbial diversity homeostasis during iron overload. At baseline, Bacteroidetes predominated in both genotypes, although the community structure differed between the WT and KO mice. After 8 weeks, Firmicutes became predominant in the WT, KO, and KDL groups, with enrichment of Oscillospiraceae possessing enzymatic capacity to degrade complex plant polysaccharides^35,36^ and contribute to fiber fermentation, including butyrate production, which supports a favorable intestinal environment for other beneficial bacteria^37^. Reduced abundance of Oscillospiraceae has been reported in obesity and metabolic disorders such as type 2 diabetes^38,39^ and has also been described in Crohn’s disease and ulcerative colitis^40^. In the present study, HID reduced the abundance of Oscillospiraceae. Comparisons of the Bacteroidetes-to-Firmicutes ratios at weeks 0 and 8 further suggested that IL-22 status influenced the relative distribution of these dominant phyla. In addition, the KD group showed an expansion of *Parabacteroides goldsteinii* (SGB.37), which, in preliminary analyses, showed a strong positive association with serum IL-22. Although typically a commensal anaerobe, *Parabacteroides goldsteinii* may behave as an opportunistic pathogen in immunocompromised hosts and has been associated with intra-abdominal infections, abscess formation, and sepsis^41^. Together, these findings position *P. goldsteinii* as a potential microbial biomarker associated with iron overload.

Furthermore, high-iron feeding was associated with metabolic reprogramming, marked by the accumulation of metabolites linked to hepatic injury and diabetogenic risk. The predicted increase in lithocholic acid (LCA) and docosahexaenoic acid (DHA) levels in the HI group may indicate the presence of two interconnected pathological axes. First, excess LCA reflects bile acid dysregulation and has been reported to promote liver injury through mitochondrial dysfunction^42^ and exacerbate intestinal inflammation via FXR-mediated signaling^43^. Second, *Parabacteroides goldsteinii* has been reported to contribute to secondary bile acid production, thereby activating the FXR/TGR5 pathway and perturbing glucose and lipid metabolism^44^. Although DHA is generally cardioprotective at physiological concentrations, supraphysiological accumulation has been reported to induce lipid peroxidation^45^ and impair fetal muscle development^46^. Concurrently, elevated fructose/glucose/galactose signatures and suppression of glycolysis/gluconeogenesis-related pathways suggest impaired glucose disposal, consistent with established mechanisms whereby iron overload promotes insulin resistance through ROS-driven inhibition of IRS-1^47^. In addition, reduced tryptophan biosynthesis and diminished indole-3-propionic acid production suggest disrupted microbiota-host metabolic crosstalk, which has the potential to aggravate metabolic inflammation. Notably, LCZ intervention largely normalized these perturbations toward CT levels, supporting its ability to counteract iron-associated metabolic dysfunction. The restoration of bile acid-related signatures and carbohydrate flux further indicated a dual modulatory role for LCZ. Together with correlation analyses showing negative associations between IL-22 and health-associated taxa and positive associations with opportunistic taxa, these findings are consistent with IL-22 functioning as a key nexus linking HID exposure, microbial remodeling and LCZ responsiveness.

The metabolic patterns observed in wild-type and IL-22 knockout mice were consistent with these findings. The WD group showed increased predicted levels of deoxycholic acid, lithocholic acid, and related derivatives, such as ketodeoxycholate, accompanied by a reduction in the predicted levels of citrulline and arachidonic acid. These shifts raise the possibility that high-iron feeding enhances secondary bile acid production, potentially through microbial community restructuring and/or modulation of host or microbial enzymatic activity, including bile acid hydrolases. As bioactive signaling molecules, bile acids can modulate lipid metabolism and inflammatory responses through the FXR and TGR5 pathways^48^, which is consistent with prior observations. In contrast, the KD group exhibited a distinct metabolic profile, characterized by higher hydrocinnamic acid levels and reduced α-muricholate levels. Notably, IL-22 deletion maintained the elevation of deoxycholic and lithocholic acids, whereas LCZ intervention attenuated these changes, suggesting that IL-22 deficiency amplified HID-associated bile acid dysregulation, whereas LCZ reversed selected abnormalities, albeit within a constrained regulatory range.

Pathway-level analyses further indicated that IL-22 knockout mice exhibited more pronounced metabolic perturbations under high iron conditions than wild-type mice. Both the WD and KD groups showed significant alterations in carbohydrate metabolism, including glycolysis, the pentose phosphate pathway, and lactose/galactose degradation. The downregulation of lactose/galactose degradation in both the WD and KD groups may be linked to the reduced abundance of probiotic Bifidobacterium species, with potential consequences for lactose utilization^49^. In parallel, reduced IPA synthesis through the *Clostridium sporogenes* pathway in the WD group may have compromised barrier-supportive mechanisms, because IPA functions as a key aryl hydrocarbon receptor (AhR) ligand^50^. The KD group exhibited additional pathway disruptions, including the downregulation of tryptophan synthesis and tyrosine degradation II, which have been associated with increased susceptibility to inflammation and autoimmunity^51^. Upregulation of nitric oxide degradation II (NO reductase) may further aggravate redox imbalance by altering NO handling at enzymatically active sites^52^. Collectively, these findings support condition-dependent pathway dysregulation driven by HID exposure and/or IL-22 deletion, followed by a partial recovery. Notably, LCZ intervention in KO mice (KDL group) restored several perturbed pathways to levels observed in the KO control group.

This study provides evidence supporting IL-22 as a key regulator of high iron diet (HID)-associated disruption of the iron metabolism-gut microbiota axis and indicates that LCZ improves iron-related indices and microbial ecological balance by modulating IL-22 signaling. By integrating serum biochemical measurements, multi-omics profiling, and genetic knockout models, the results delineated a diet-IL-22-microbiota-metabolic dysfunction regulatory network and highlighted potential targets and intervention strategies relevant to nutritional iron overload. This study had some limitations. The metabolite and pathway analyses presented in this study were based on functional predictions derived from metagenomic data rather than direct metabolomics measurements. Future research using targeted or untargeted metabolomics methods is necessary to verify these predicted changes and establish a direct connection between microbial functional potential and metabolic phenotypes.

This study demonstrated that LCZ intervention ameliorated the dysregulation of iron-related indices under high-iron dietary conditions, associated with IL-22-dependent responses. LCZ attenuated iron overload-associated alterations, including partial restoration of total iron-binding capacity (TIBC) and suppression of elevated IL-22 levels, and exhibited minimal effects under control conditions. Metagenomic profiling further indicated that LCZ remodels gut microbial ecology by increasing the relative abundance of taxa commonly linked to gut health, including *Bifidobacterium* species (e.g., *Bifidobacterium animalis*), and reducing the enrichment of opportunistic taxa, such as *Parabacteroides goldsteinii*, along with the normalization of bile acid and glucose metabolism-related functional signatures. Genetic knockout experiments supported IL-22 as a mediator required for LCZ-associated modulation of iron indices in iron overload. Collectively, these findings support a probiotic-based approach targeting IL-22–linked immune-microbiota interactions as a potential strategy to mitigate the adverse effects associated with excessive dietary iron.

## Methods

### Materials and reagents

The probiotic strain *Lacticaseibacillus casei Zhang* (LCZ; GenBank accession no. CP001084) was obtained from the Lactic Acid Bacteria Cell Collection of the Key Laboratory of Dairy Biotechnology and Engineering at Inner Mongolia Agricultural University, China. A high-iron diet (HID; 500 ppm iron, TP0452M) and the corresponding control diet (45 ppm iron, TP0452) were purchased from Trophic Animal Feed High-Tech Co., Ltd. (Nantong, China). All diets were SPF-grade, derived from the same production lot (sterilization batch no. 21050419), and handled under SPF conditions throughout the study. Commercial assay kits were used according to the manufacturer’s instructions to measure serum iron (Beijing Kemeidongya Biotechnology Co., Ltd.), total iron-binding capacity (TIBC; Beijing Leadman Biochemistry Technology Co., Ltd.), and multiplex immunoassay analytes (ProcartaPlex™, Thermo Fisher Scientific, USA).

### Animal experiments

Eight-week-old male C57BL/6N mice were purchased from Beijing Huafukang Bioscience Co., Ltd. (Animal Production License No. SYXK (Bejing) 2019-0022). IL-22 knockout mice on a C57BL/6N background, generated using CRISPR/Cas9 technology, were obtained from Cyagen Model Organisms Research Center (Taicang, China) (Animal Production License No.: SCXK (Su) 2018-0003) and then transferred to Beijing Huafukang for experimental use. All animals were maintained under specific pathogen-free (SPF) conditions at 22–24 °C and 40–50% relative humidity, on a 12 h light-dark cycle, with ad libitum access to chow and water. The mice were acclimated for one week before the initiation of the interventions.

As shown in Fig. 1A, after acclimation, thirty-two 8-week-old SPF-grade C57BL/6N mice were randomly assigned to four groups (*n* = 8 per group): CT (control diet with oral gavage of 0.1 mL/day saline), CTL (control diet with oral gavage of 0.1 mL/day LCZ), HI (high-iron diet with oral gavage of 0.1 mL/day saline), and HIL (high-iron diet with oral gavage of 0.1 mL/day LCZ). Mice in the CTL and HIL groups received LCZ at 2 × 10^9^ CFU/mL concentration.

In the validation experiment, 15 IL-22 knockout (KO) mice and 10 wild-type (WT) mice with a matched genetic background were acclimated for one week and then allocated as follows. KO mice were randomized into three groups: KO (control diet with oral gavage of 0.1 mL/day saline), KD (high-iron diet with oral gavage of 0.1 mL/day saline), and KDL (high-iron diet with oral gavage of 0.1 mL/day LCZ). WT mice were allocated into two groups: WT (control diet with oral gavage of 0.1 mL/day saline) and WD (high iron diet with oral gavage of 0.1 mL/day saline). The LCZ dosage in the KDL group was the same as that used in the primary experiments (Fig. 4A).

All experiments were conducted over eight weeks. Body weight and food intake were monitored and recorded weekly. At the end of the experiment, mice were anesthetized by intraperitoneal injection of 1% sodium pentobarbital at a dose of 50 mg/kg body weight. Anesthesia depth was confirmed by the absence of a pedal withdrawal reflex. Blood was collected via retro-orbital bleeding while the mice were under deep anesthesia. After blood collection, mice were euthanized by cervical dislocation under deep anesthesia. All efforts were made to minimize suffering. Blood samples were used for serum isolation and subsequent biochemical and immunological analysis. Fecal samples were collected at baseline (week 0) and at study completion (week 8), immediately snap-frozen, and stored at −80 °C until subsequent analyses.

### Ethical approval

All animal procedures were conducted in accordance with the Guidelines for Care and Use of Laboratory Animals of Inner Mongolia Agricultural University (Permit No: SYXK-2020-0002) and were approved by the Animal Ethics Committee of Inner Mongolia Agricultural University (No: NND2023105).

### Determination of organ coefficients and serum iron-related revealed

After euthanasia and dissection, the livers and spleens were excised and weighed. The organ coefficients were calculated as the ratio of organ weight (mg) to body weight (g). Serum iron (SI) and total iron-binding capacity (TIBC) were quantified using the Ferene method on a Beckman Coulter AU 480 analyzer (Beckman Coulter, Inc., USA) according to Derman et al.^53^. The linear detection range was 0–179 μmol/L for both SI and TIBC.

### Measurement of serum immune factors

Serum immune markers (IL-22, IL-1a, IL-5, IL-13, IL-18, IL-23, and IL-27) were quantified using xMAP technology on a Luminex 200™ system. Commercial MILLIPLEX® MAP kits (EMD Millipore) were used according to the manufacturer’s protocol. Standard curves were generated using the instrument software, and analyte concentrations were determined by interpolation from the corresponding curves. All results are expressed as pg/mL.

### DNA extraction and metagenomic sequencing

Metagenomic DNA was extracted from fecal samples using the QIAamp Fast DNA Stool Mini Kit (Qiagen, Hilden, Germany) following the manufacturer’s instructions. DNA integrity and quality were assessed using agarose gel electrophoresis, NanoDrop spectrophotometry (260/280 ratio, 1.8–2.0), and Qubit® fluorometry. Only samples meeting the predefined quality criteria (DNA concentration >20 ng/μL) were subjected to library construction. Sequencing libraries were prepared using 1 μg of DNA per sample with the NEBNext® Ultra™ DNA Library Prep Kit for Illumina sequencing. Briefly, DNA was fragmented by sonication to an average insert size of ~350 bp, followed by end repair, A-tailing, Illumina adapter ligation, size selection and PCR amplification. Libraries were purified using AMPure XP beads, evaluated using an Agilent Bioanalyzer, and quantified using real-time PCR. Qualified libraries were indexed, clustered on a cBot system, and sequenced on an Illumina NovaSeq/HiSeq platform to generate paired-end read sequences.

### Metagenomic assembly, binning, and dereplication

Metagenomic reads from each sample were assembled de novo into contigs using MEGAHIT (v1.1.3). Contigs exceeding 2000 bp in length were extracted for genome binning. Metagenome-assembled genomes (MAGs) were reconstructed from the contigs using MetaBAT2 (v2.12.1) under default parameters^54^. To generate coverage profiles, reads from each sample were mapped back to their respective assembled contigs with BWA-MEM (v0.7.17)^55^, and contig coverage depths were calculated using SAMtools (v1.9)^56^ and the jgi_summarize_bam_contig_depths script from the MetaBAT2 package.

The quality of the reconstructed MAGs was assessed using CheckM (v1.0.7)^57^ based on estimates of genome completeness and contamination. MAGs were classified as high-quality (≥80% completeness, ≤5% contamination), medium-quality (≥70% completeness, ≤10% contamination), or partial-quality (≥50% completeness, ≤5% contamination) according to established standards. These MAGs were subsequently dereplicated using dRep (v3.2.0)^58^ to cluster redundant genomes and select a single, highest-quality representative from each species-level cluster, applying thresholds of 95% average nucleotide identity (-pa 0.95), and 95% average alignment fraction (-sa 0.95).

### Taxonomic annotation and functional profiling

Species-level genome bins (SGBs) were taxonomically classified using Kraken2 against the NCBI non-redundant nucleotide database, and their relative abundances were quantified as RPKM using CoverM under stringent parameters (-min-read-percent-identity 0.95 -min-covered-fraction 0.4). Functionally, open reading frames were predicted and assigned to KEGG Orthology (KO) terms. Leveraging these KOs, metabolic modules were delineated using Omixer-RPM (-c 0.66), and gut metabolic modules (GMMs) were predicted from the MetaCyc database.

### Statistical analysis

Statistical analyses were performed using non-parametric methods. The Kruskal–Wallis test was applied for comparisons among multiple groups, and the Mann–Whitney U test was used for two-group comparisons. Data were visualized using R software (version 3.6.1; https://www.r-project.org/) and GraphPad Prism 9 software. The results are presented as mean ± standard deviation (SD). In addition, specialized R packages were used for ecological and multivariate analyses, including vegan for community ecology metrics, mixOmics for multivariate modeling, and ggplot2 with ggpubr for graphical representation. Alpha diversity indices (e.g., Shannon and Simpson) were calculated, and multivariate patterns were explored using principal component analysis (PCA) and partial least squares discriminant analysis (PLS-DA). Associations were evaluated using Pearson’s correlation coefficients. Where applicable, adjustments for multiple comparisons were performed.

## Supplementary information

Supplementary information

## Data Availability

The datasets generated and/or analyzed during the current study are available in the National Genomics Data Center (NGDC) repository. The data can be accessed using accession number CRA039560 (https://share.cncb.ac.cn/Xkv1xhku).
