# Supplementary material for: Lacticaseibacillus casei Zhang modulates iron homeostasis in iron-overloaded mice via an IL-22-dependent pathways
Source: NPJ Sci Food. 2026 May 26;10:253. doi: 10.1038/s41538-026-00899-0 (PMC13487296; doi:10.1038/s41538-026-00899-0)
Supplement: Supplementary file 1 [file 41538_2026_899_MOESM1_ESM.pdf]

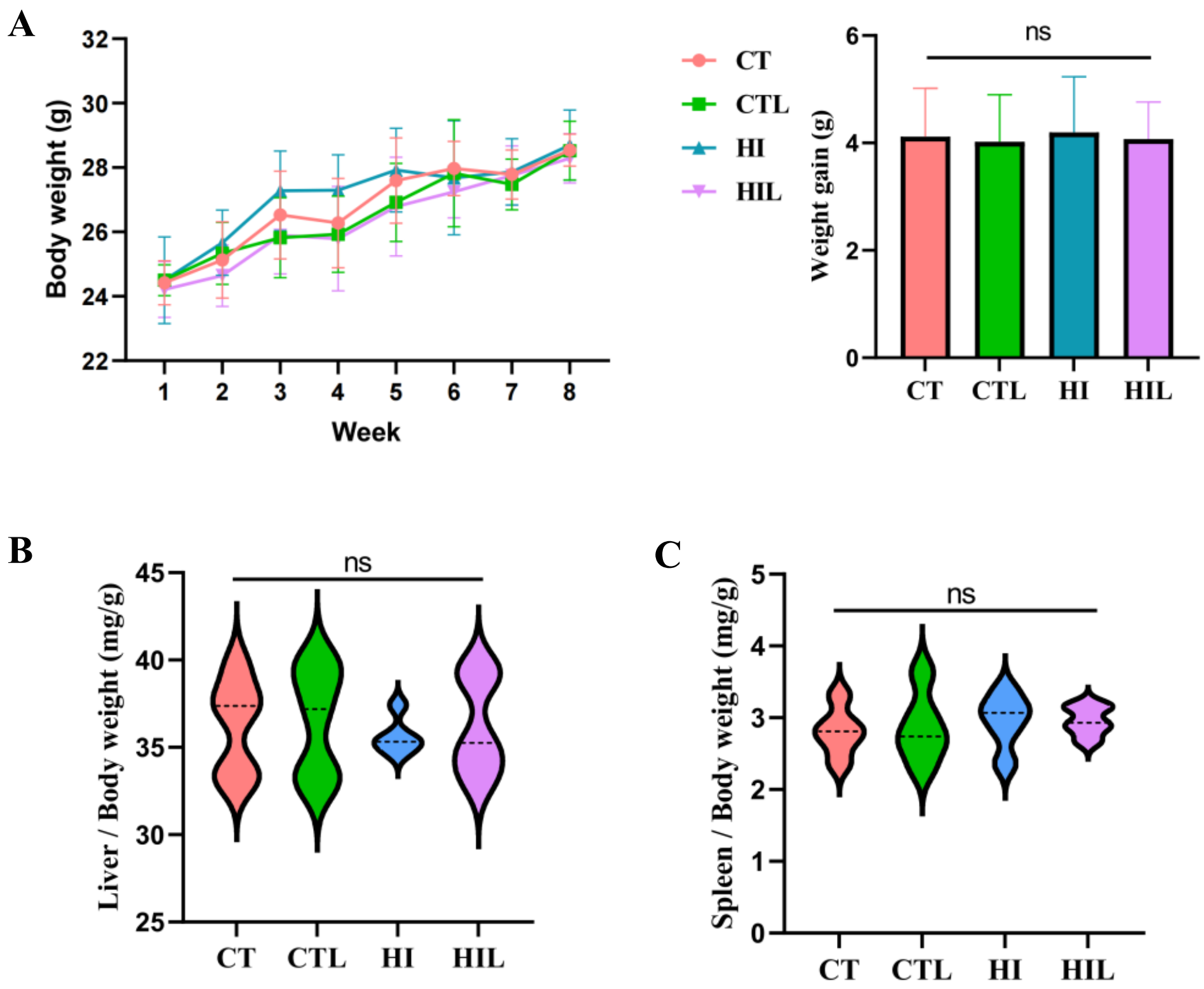

Supplementary Figure 1. (A) Body weight. (B) Liver organ coefficients (B) spleen organ coefficients. No significant differences were observed in the liver or spleen organ coefficients between the groups. Error bars represent SD.
